# Repeated acute coronary syndrome caused by a mind-bending mural thrombus in ascending aorta: a case report and review of the literature

**DOI:** 10.1186/s12872-024-03956-2

**Published:** 2024-05-29

**Authors:** Hanxuan Liu, Zhangjie Yu, Ying Xu, Yan Zhou, Juntao Yang, Yinyin Qiu, Yangbo Xing, Fang Peng, Weiliang Tang

**Affiliations:** 1 School of Medicine, Shaoxing University, Shaoxing City 312000, Zhejiang Province China; 2 Department of Cardiology, Shaoxing People’s Hospital, NO. 568 North Zhongxing Road, Yuecheng district, Shaoxing City 312000, Zhejiang Province China

**Keywords:** Ascending aortic thrombus, Primary aortic mural thrombus, Acute coronary syndrome, Coronary artery embolism

## Abstract

**Background:**

Acute coronary syndrome due to coronary artery embolism in the setting of ascending aortic thrombus is an uncommon condition, even rarer when there is no aortic pathology such as aneurysm, severe atherosclerosis, aortic dissection, or thrombophilia (whether inherited or acquired).

**Case presentation:**

We report a case of a 58-year-old male presented with acute chest pain, electrocardiogram showing non-ST-elevation acute coronary syndrome. The computed tomography angiography of coronary artery revealed a mural thrombus in the proximal part of ascending aorta, located above the left coronary artery ostium, without any aortic pathologies. With the exception of hypertension and cigarette smoking, no other risk factors were identified in this patient that may increase the risk of thrombosis. Given the life-threatening risk of interventional therapy and surgery, the patient determinedly opted for anticoagulant and dual antiplatelet therapy. Then he experienced the reoccurrence of chest pain after 6-day treatment, progressed to anterior and inferior ST-segment elevation myocardial infarction. Coronary artery embolism originating from the ascending aortic thrombus was suspected. Considering the hemodynamic instability of the patient, the medical treatment was continued and bridged to warfarin and aspirin after discharge. Follow-up computed tomography angiography at 6 months showed no obstruction in coronary artery and complete resolution of the thrombus. No thromboembolic events occurred henceforward.

**Conclusions:**

Acute coronary syndrome could be a manifestation of secondary coronary embolism due to ascending aortic thrombus. Currently, there is no standardized guideline for the treatment of aortic mural thrombus, individualized treatment is recommended. When surgical therapy is not applicable for the patient, anticoagulation and dual antiplatelet treatment are alternative treatments that may successfully lead to the resolution of the aortic thrombus.

**Supplementary Information:**

The online version contains supplementary material available at 10.1186/s12872-024-03956-2.

## Background

Acute coronary syndrome (ACS) is commonly attributable to coronary atherosclerotic plaque rupture, resulting in thrombosis and obstruction in coronary artery. Nevertheless, coronary artery embolism (CAE), as an infrequent nonatherosclerotic cause of ACS, should also be taken into account. It was reported that the incidence of acute myocardial infarction caused by CAE is 2.9% [1].One systemic review demonstrated that the most commonly documented etiologies of CAE included infective endocarditis (22.4%), atrial fibrillation (17.0%), and prosthetic heart valve thrombosis (16.3%), only one CAE case was caused by an ascending aortic thrombus (0.6%) [2]. Ascending aortic thrombus is usually associated with aortic pathologies, including aortic aneurysm, severe aortic atherosclerosis, and aortic dissection [3]. Ascending aortic thrombus in the absence of the lesions above is very uncommon. This report presents a rare case of the ACS secondary to a mural thrombus sited in a normal ascending aorta, ultimately well-treated with anticoagulation and antiplatelet agents.

## Case presentation

**Fig. 1:**
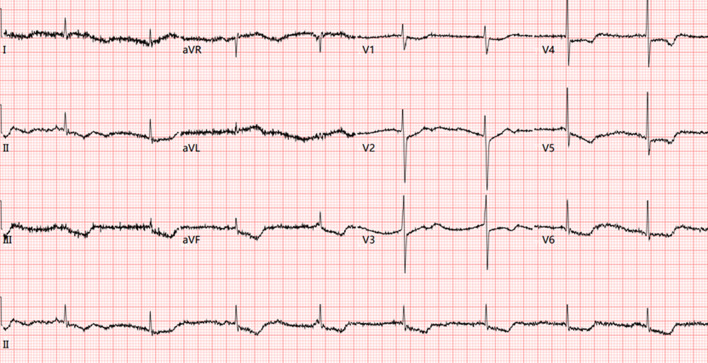
Emergency room 12-Lead Electrocardiogram presenting ST-segment depression in leads II, III, AVF and V1-V6, indicating acute non-STEMI

A 58-year-old male presented to our emergency department with excruciating chest pain lasting for over 30 min. He had a smoking addiction (over 300 packs of cigarettes per year for more than 20 year) and a history of hypertension that was well-controlled with antihypertensive drugs (including nifedipine, telmisartan and hydrochlorothiazide), with no previous history of atrial fibrillation, valvular diseases or thromboembolic events. On admission, his blood pressure was too low to detect, and the oxygen saturation level in atmospheric air dropped to 90.1%. The initial electrocardiogram (ECG) showed persistent ST-segment depression in leads II, III, AVF and V1-V6, indicating ongoing massive myocardial ischemia and acute non-ST elevation myocardial infarction (non-STEMI) (Fig. 1). The serum troponin I (TnI) level increased to 2.46 ng/mL. Echocardiography showed left ventricular dyskinesia with an enlarged left atrium and an ejection fraction of 27%, negative for endocarditis, valve abnormalities, or any thrombus in the cardiac chambers (Supplementary File1 and File 2). Based on the latest guideline for the management of ACS [4], the patient was advised for invasive coronary angiography and emergency percutaneous coronary intervention, which was rejected by the patient’s dependents for the high risk of intraoperative adverse events. Emergency treatments were immediately performed including a loading dose of aspirin and clopidogrel, dopamine boost, norepinephrine and low molecular weight heparin (LMWH).

**Fig. 2:**
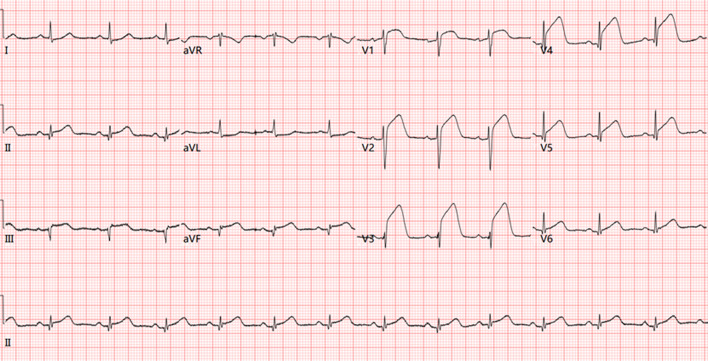
Bedside Electrocardiogram after the reoccurrence of chest pain shows ST-segment elevation in leads II, III, AVF and V1-V5, with Q waves in leads I II, III, AVF, indicating acute ST-segment elevation myocardial infarction in extensive anterior and inferior walls

**Fig. 3:**
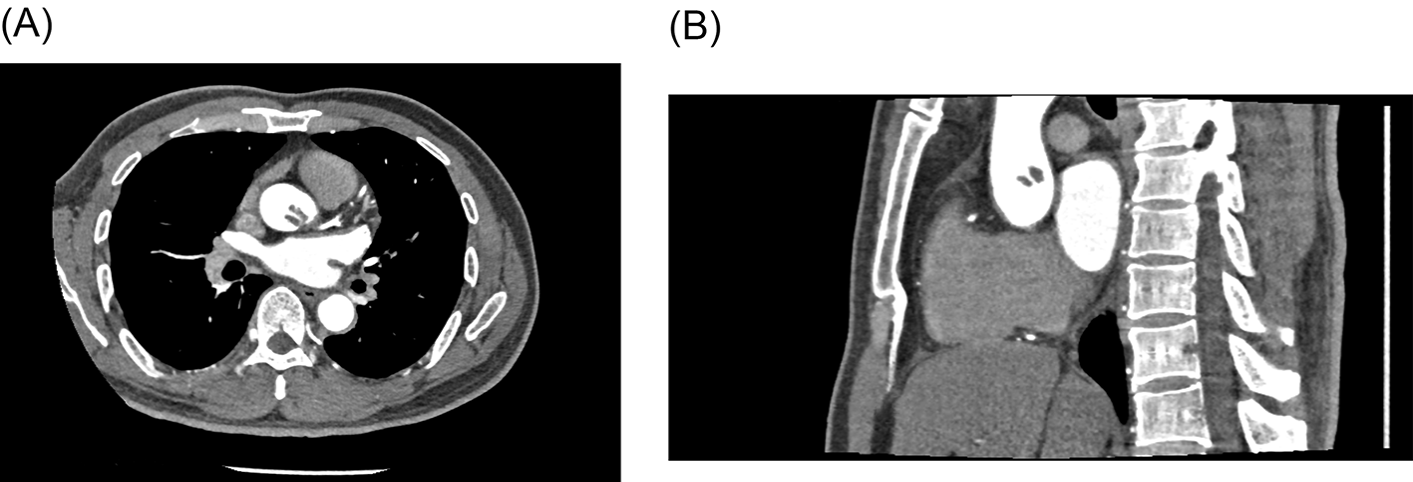
Coronary CTA images in (**A**) axial and (**B**) sagittal planes showing a lobulated filling defect suggestive of a thrombus, located in the proximal portion of ascending aorta. The thrombus occurs just above the left coronary ostium, closed to the sinotubular junction and the sinus of Valsalva. No abnormality was found in the coronary artery

**Fig. 4:**
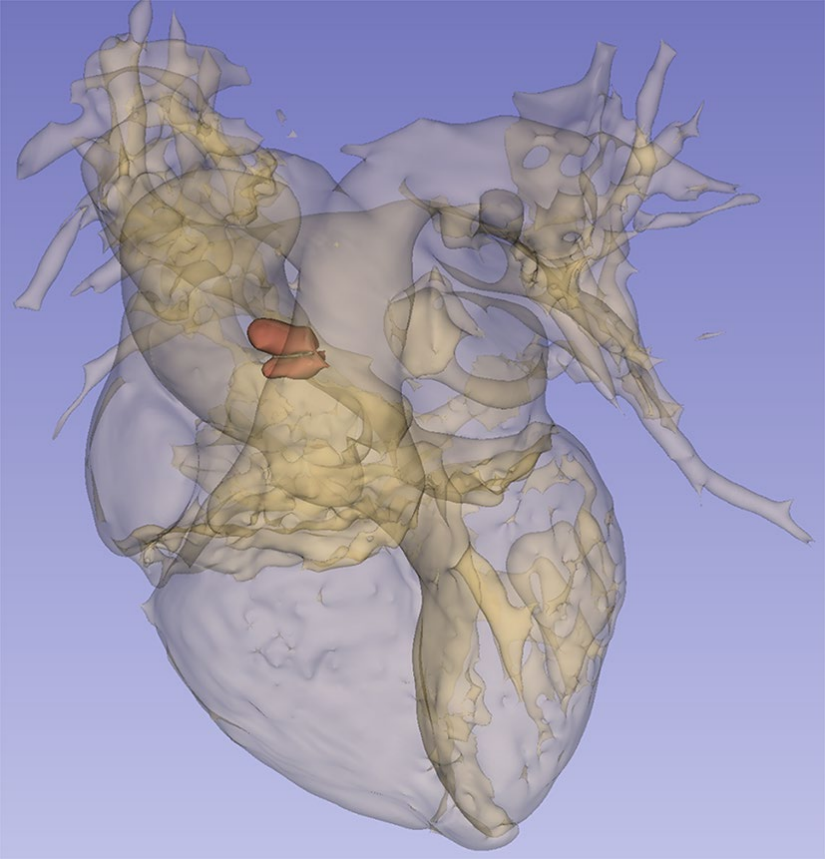
3D reconstruction based on coronary CTA revealing the presence of a large thrombus in the proximal portion of the ascending aorta, measuring 1.5 × 1.1 × 0.9 cm. Red structure indicates the thrombus

**Fig. 5:**
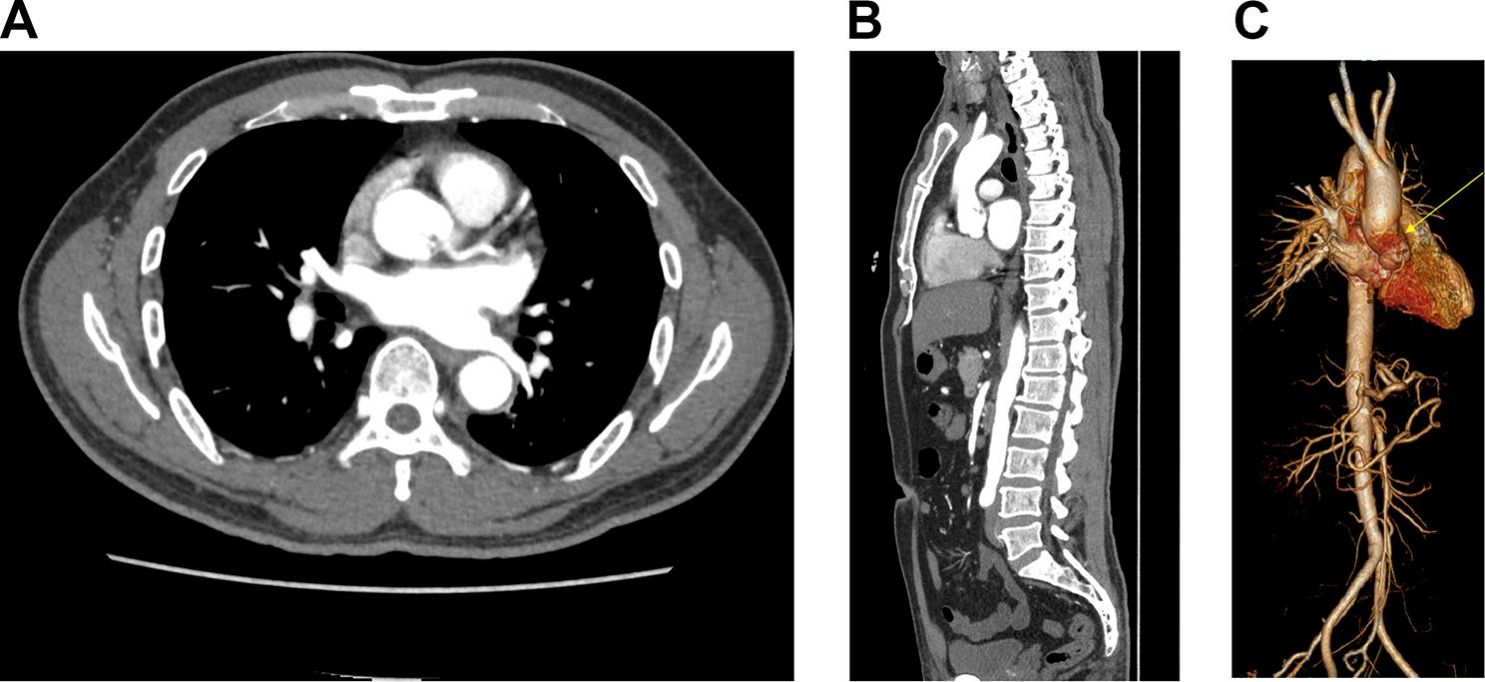
(**A**) and (**B**): The pursued CTA of aorta showed patency of coronary artery. The original thrombus decreased in size and became indistinct at the edges after the short-term anticoagulant and dual antiplatelet therapy. (**C**) Three-dimensional reconstruction of the aorta. Yellow arrow indicates the dissolving thrombus

**Fig. 6:**
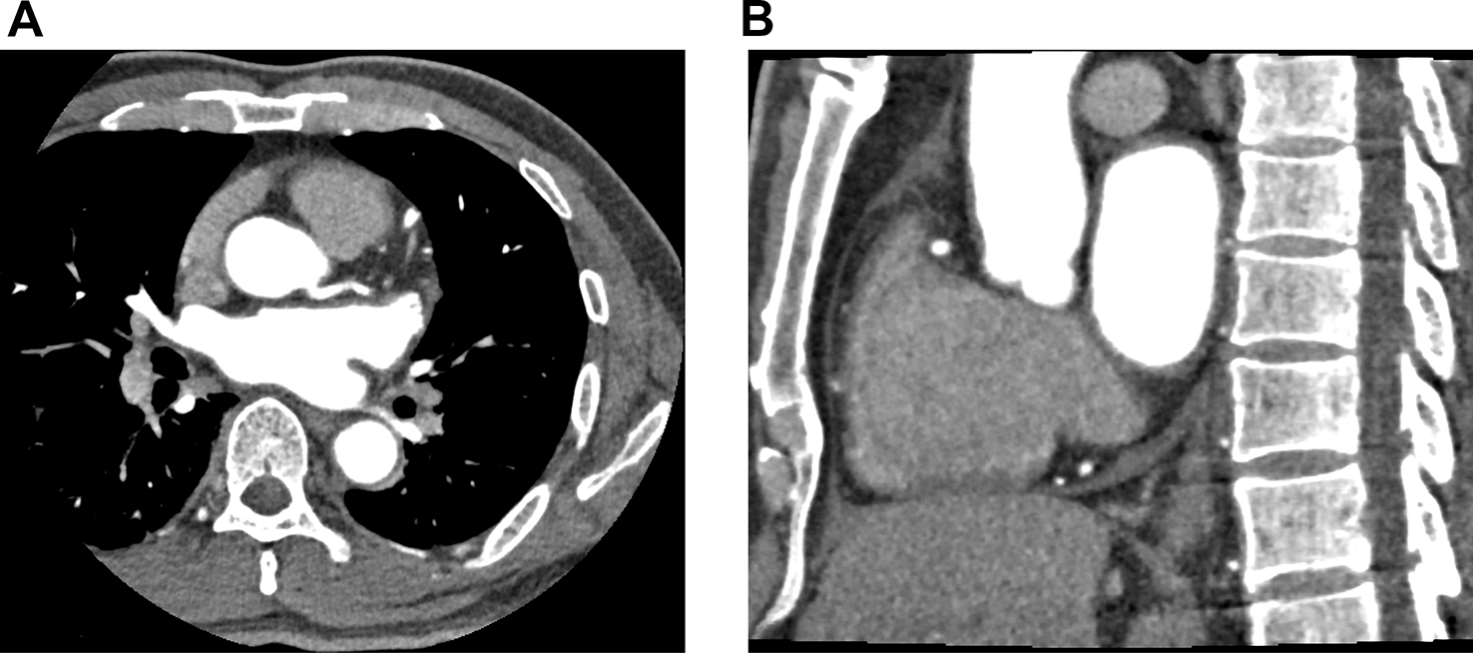
(**A**) and (**B**): Repeated coronary CTA before discharge showed no obvious abnormalities

**Fig. 7:**
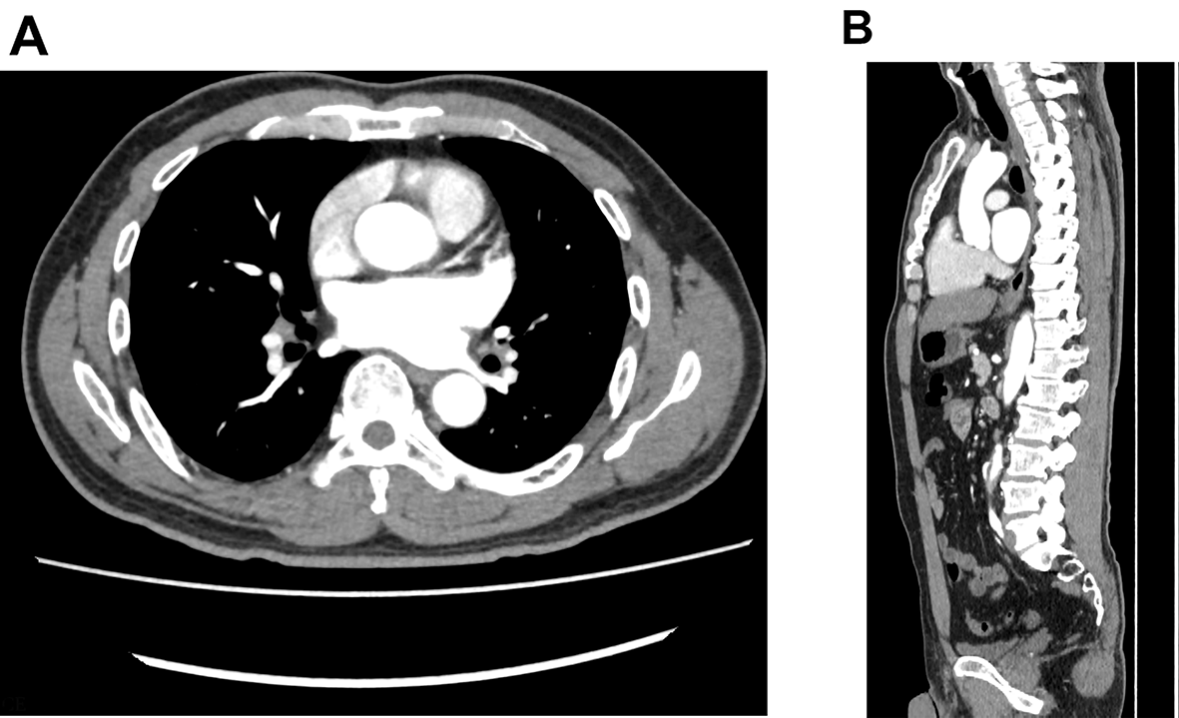
(**A**) and (**B**): Follow-up CTA at 6 months demonstrating complete resolution of the previous thrombus

The patient was transferred to the department of cardiology after the symptoms were palliated. Laboratory data showed a high D-dimer level (2.25 µg/mL). Blood cell count, tumor markers antinuclear antibody and blood lipid profile were normal. The computed tomography angiography (CTA) of coronary artery demonstrated a completely normal coronary artery, with no obstructions or stenosis (Fig. 3). Unexpectedly, the CTA identified a lobulated mass in the proximal part of ascending aorta, located above the left coronary ostium, measuring 1.5 × 1.1 × 0.9 cm, suggestive for a mural thrombus in the ascending aorta (Fig. 4). The pursued CTA of aorta obtained the consistent result that the coronary artery showed no obvious abnormality, and that no signs of atherosclerotic deposits, aneurysms or aortic dissection were found in the ascending aorta (Fig. 5). The patient had received short-term anticoagulant and antiplatelet therapy before receiving the aortic CTA, which showed that the original thrombus decreased in size and became indistinct at the edges. His Society of Thoracic Surgery score and European System for Cardiac Operative Risk Evaluation (EuroSCORE) II were 59.8% and 20.81% respectively, for isolated coronary artery bypass grafting (CABG). Considering the life-threatening risk of interventional therapy and surgery, the patient was determined on conservative treatment. Therefore, dual antiplatelet and anticoagulant therapy (aspirin 100 mg, clopidogrel 75 mg and LMWH) were still administered, which subsequently relieved his chest pain. Echocardiography revealed LVEF of 45%, and left atrial diameter (LAD) 32 mm, left ventricular internal dimension at end-diastole (LVIDd) 50 mm (shown in Supplementary Fig. 1A and Fig. 1B in the revised manuscript). However, after the 6 days of conservative treatment, the patient was attacked by a recurrence of chest pain and became hemodynamically unstable. Bedside ECG showed ST segment elevation in precordial leads V1 to V5 and limb leads II, III, AVF, indicating extensive anterior and inferior myocardial infarction (Fig. 2). Serum troponin I level increased significantly to 36.17ng/mL. CAE originating from the ascending aortic thrombus accounting for the ACS was suspected. In such condition, coronary intervention carries a hazard of catheter-induced thrombus dislodgement resulting in life-threatening thromboembolic events, including but not limited to acute coronary occlusion and cerebral embolism. As the thrombus had been present for several days (over 24 h), it already passed the time window for intravenous thrombolysis. Therefore, intravenous thrombolytic therapy not only has little effect on resolving such a subacute thrombus but also contributes to bleeding tendencies. Even worse, the subsequent dissolution and dislodgement of the thrombus might pose a risk of secondary arterial embolism. In order to properly get rid of the thrombus, the patient was referred to the department of cardiovascular surgery for surgical thrombectomy. However, considering the huge traumatic stress that the surgery brings, as well as the possible risk of the thrombus dislodgement during operation, the patient rejected the surgical thrombectomy in the end. He continued the conservative treatment consisting of Vasodilators, dual antiplatelet and anticoagulant agents for the remaining days of the hospital stay, which fortunately alleviated the chest pain. Afterwards, the serum troponin I turned to normal level and repeat coronary CTA showed complete resolution of the lobulated thrombus without any abnormality in the coronary artery (Fig. 6). The patient was discharged with warfarin (2.5 mg) and aspirin(100 mg), followed up biweekly to ensure the international normalized ratio (INR) ranged between 2.0 and 3.0. Repeat echocardiography at 1month indicated his cardiac function was basically recovered (shown in Supplementary Fig. 2A and Fig. 2B). Follow-up CTA at 6 months revealed no thrombus in the aortic root or ascending aorta (Fig. 7). No thromboembolic events occurred henceforward.

## Discussion

ACS due to an aortic mural thrombus (AMT) sited in the ascending aorta is a distinctive and uncommon cause of acute myocardial infarction. In many cases, AMT is usually associated with aortic pathologies, including severe atherosclerosis, vasculitis, aneurysm and dissection of the aorta. Primary aortic mural thrombosis (PAMT), defined as a thrombus attached to the aortic wall in the absence of any atherosclerotic or aneurysm disease in the aorta, is a relatively rare entity. Machleder et al. reported an incidence rate of 0.45% for non-aneurysmal AMT based on a consecutive series of 10,671 autopsies, none of which involves ascending aorta [5]. In addition, the proximal portion of the ascending aorta is an uncommon site for PAMT. Existing literature acknowledged that the descending thoracic aorta was the most common location of the AMT (38%), while the ascending aorta were the least common location (12%) [6]. Verma et al. documented 19 patients of PAMT manifested as peripheral embolic events, of whom only 2 patients had a thrombus located in the ascending aorta [7].

The presented case has attracted much interest because of the infrequent manifestation of ACS caused by an ascending aortic thrombus. Most cases of ascending AMT are clinically silent, usually diagnosed after an embolic event or occasionally identified through echocardiography and CTA [8]. The typical embolic events caused by AMT include ischemic stroke, visceral and distal embolization, while the coronary events are relatively rare [6]. What’s unexpected, our patient underwent repeated ACS in his hospital stay. As reported in previous cases, both thrombi in the ascending aorta originating from the ostium of coronary artery and an aortic root thrombus extending into the coronary sinus of Valsalva can account for ACS [9, 10]. The occurrence of ACS could arise from the distal CAE caused by the detachment of the aortic thrombus. Ascending aortic thrombus occluding the coronary ostium is also a possible condition. The differential diagnosis relying on effective investigation seems rather critical. Though transesophageal echocardiography (TEE) can precisely localize aortic thrombi and atherosclerosis, TEE still has limitations in presenting the ascending aorta and the proximal aortic arch due to the interference of the trachea, making CTA a superior examination to detect ascending aortic thrombus [8]. Coronary CTA possess high diagnostic value in excluding significant coronary artery disease (CAD) [11]. As the first-line anatomic test, Coronary CTA could reduce the total costs of diagnosis in stable CAD patients by avoiding invasive coronary angiography and hospitalization [12]. We ultimately ruled out the cardiogenic thrombus resulting in ACS, for no evidence of atrial fibrillation and left ventricular thrombus was revealed in the CTA. Then thorough investigations about the etiologies of ascending aortic thrombosis were planned on this patient.

Although the pathophysiological mechanism of an ascending aortic mural thrombosis has not been fully explored, the principle of Virchow’s triad for thrombogenesis remains closely related. Virchow’s triad describes the three factors related to thrombosis: hemodynamics (fluid stasis), endothelial injury (associated with foreign materials), and hypercoagulability (abnormal blood chemistry) [13]. The high-flow environment of the ascending aorta, characterized by the large blood flow, the high blood pressure and high shear stress, theoretically serves as a protective mechanism against stasis and thrombosis, and contributes to the scarcity of mural thrombi at the proximal ascending aorta. Also, the helical blood flow in the ascending aorta protects the aortic wall from atherosclerosis, thrombus formation, and intimal proliferation [14]. On the other hand, the Valsalva sinuses in the aortic root complex has been reported to alleviate the high sheer stress of the aortic root. The Valsalva sinuses create a space between the aortic valve leaflets and the aortic wall. As the valve leaflets move towards the aortic wall in the early systole, the space make it possible for blood to flow along the sinotubular junction and enters the Valsalva sinuses, facilitating the eddy current development and promoting thrombosis in the ascending aorta [15]. In our case, the thrombus was located just around the sinotubular junction and the sinus of Valsalva, somehow making sense of the ascending aortic thrombosis in the patient. In addition to hemodynamics, endothelial injury and hypercoagulable states are closely associated with aortic thrombosis. Prior studies indicated that aortic endothelial injury could arise from prosthetic aortic valve implantation, aortic stent implantation, open aortic surgery, aortitis and infective endocarditis, potentially leading to thrombus-related CAE [16–20]. Therefore, actively searching for evidence of hypercoagulative conditions is our clinical strategy to determine the probable causes of ascending aortic thrombosis in a normal aorta. The hypercoagulable states that have been reported to be associated with the pathogenesis of ascending aortic thrombi include active cancer, inherited thrombophilia, thrombocythemia, polycythemia, antiphospholipid syndrome, systemic infectious diseases [5, 21–26]. However, in our case, neither associations with aortic endothelial injury and aortic pathologies such as aortic atherosclerosis, aneurysm, or dissection were found through CTA, nor were any signs indicating risk factors for the aforementioned hypercoagulable states observed during post-admission examinations. Predisposing factors for aortic thrombosis include old age, diabetes, hypertension, hyperlipidemia, smoking addiction, trauma, oral contraceptive use, hormone replacement therapy, and use of exogenous steroids [7]. Our patient unexpectedly has no other marked predisposing factors but only history of hypertension and smoking. Since hypertension was well controlled, the possible explanation is a transient hypercoagulable state induced by smoking. Also, nicotine in tobacco can cause to vascular endothelial injury, increasing the risk of aortic thrombosis. But the exact cause of thrombosis in the ascending aorta remains mysterious. The interaction of Virchow’s triad potentially led to the ascending aortic thrombosis.

A systematic review indicates that ACS due to coronary embolism has a worse prognosis than atherosclerotic ACS [2], for the former type has larger myocardial infarct areas. Early differential diagnosis and coronary revascularization are crucial. One must rule out a thrombus within an aneurysm and aortic atherosclerotic thrombus, for the different therapeutic avenues. Aortic mural thrombi associated with aneurysmal formation are most sited in the abdominal aorta, known as intraluminal thrombus (ILT). ILT adheres firmly to the aortic wall, so it’s rare the ever-growing ILT leads to distal occlusion. Anticoagulation is notoriously difficult to reverse in the event of hemorrhage. Currently, neither antiplatelet nor anticoagulant therapy is clinically used except aspirin recommended as secondary prevention for abdominal aortic aneurysm-induced cardiovascular diseases [27]. Aortic atherosclerotic thrombosis involves thrombus building up on complex or ulcerated aortic plaques. The present evidence supports that dual antiplatelet therapy or moderate intensity anticoagulation with warfarin as secondary prevention of aortic atheroma could decrease the recurrence of thromboembolic stroke [28]. There is no consensus or properly evolved guidelines about the management of ascending aortic thrombus because of its scarcity, so the management of AMT rely much on clinical experience. Treatment options reported so far include anticoagulation, antiplatelet therapy, the combination of anticoagulation and antiplatelet agents, thrombolysis, endovascular stent graft placement, and surgical therapy. Individualized treatment is recommended, taking into consideration factors including the size, location, mobility and implantation base of the thrombus, hemodynamic stability of the patient, and the medical standard of the hospital. Surgery is the recommended treatment for AMT in a normal or mildly atherosclerotic aorta and thrombus located in the ascending aorta, with lower incidence of recurrent thrombus, thromboembolic complication, and limb loss [6]. As for thrombus located in the aortic arch, descending aorta, and abdominal aorta, endovascular treatment or medical treatment are the preferred option [29]. Aortic mural thrombi are classified as sessile and pedunculated based on the implantation base. For large and pedunculated thrombi bear a higher risk of secondary embolism, surgical treatment may obtain better clinical results [30]. In contrast, small and sessile thrombi are appropriate for anticoagulation [31]. Thrombi over 1 cm in diameter were reported with higher risks of embolic events [32]. Previous studies postulated that anticoagulation may lysing the thin attachment site of the pedunculated thrombus before lysing the thrombus itself, thus triggering the subsequent embolic events [31]. Laperche et al. reported 23 patients with isolated ascending aortic arch thrombus, among whom 17 patients were treated with anticoagulants, and the aortic arch thrombi in 11 patients (92%) disappeared from 3 days to 6 months after the initial embolic event, indicating the therapeutic potential of anticoagulants in the management of ascending aortic thrombus. Choukron et al. recommended anticoagulation as initial therapy and resorting to surgical thrombectomy only when anticoagulation proves ineffective [33]. Some cases also reported successful resolution of aortic thrombi under the combination of anticoagulation and antiplatelet therapy, but it brings an increased tendency of bleeding [34]. Currently, the optimal drug, dose, and duration of anticoagulation therapy for ascending aortic thrombus have not been standardized, and no study has assessed direct oral anticoagulants in aortic thrombus so far [28]. We referred to the management for left ventricular thrombus and prescribed the patient warfarin with stable INR (within the range of 2.0–3.0) [35]. Endovascular stent graft is less invasive than surgery, also possibly decreasing the size of residual aortic thrombus with a reduced risk of recurrent embolization in comparison to anticoagulation. But when dealing with ascending aortic thrombus, endovascular treatment may pose the risk of dislodging the thrombus during the stent placement, giving rise to fatal embolism [7]. Thrombolysis, though infrequently performed in managing aortic thrombus, has also provided successful treatments in some cases [36]. Conventional surgery can remove the aortic thrombus once for all and avoid recurrent embolism, but as a more invasive choice, it involves sternotomy procedure, cardiopulmonary bypass, deep hypothermia and a subsequent series of post-operative complications, including arrhythmias and even post-thrombectomy mortality [37]. Pre-operative hemodynamic instability is usually associated with the increased risks of surgical mortality. Considering our patient’s hemodynamic instability and his strong preference for conservative treatment, we determined on a safer management, which consisted of anticoagulant and dual antiplatelet agents. Fortunately, the patient’s symptoms and quality of life were all improved at the six-month follow-up.

## Conclusion

ACS due to coronary artery embolism in the setting of a mural thrombus sited in a normal ascending aorta is rather rare. This case report has attempted to elaborate the potential mechanisms of ascending aortic thrombosis from the perspective of the well-known Virchow’s triad. Active hypercoagulable investigations on the patients are essential. Our patient was successfully treated with antiplatelet and anticoagulant therapy, it should be noted that AMT sited in the ascending aorta is a hazardous condition. When surgery therapy was unavailable or inapplicable, antiplatelet and anticoagulant therapy could bring a chance of resolving ascending mural thrombi. However, it also comes with possibilities of secondary embolic events, including CAE as one of the worst conditions.

### Electronic supplementary material

Below is the link to the electronic supplementary material.

Supplementary Material 1

Supplementary Material 2

Supplementary Material 3

Supplementary Material 4

Supplementary Material 5

Supplementary Material 6

## Data Availability

All data generated or analysed during this study are included in this article.

## Abbreviations

ACS: Acute Coronary Syndrome
CAE: Coronary Artery Embolism
ECG: Electrocardiogram
non-STEMI: non-ST elevation myocardial infarction
TnI: Troponin I
LMWH: Low Molecular Weight Heparin
CTA: Computed Tomography Angiography
CABG: Coronary Artery Bypass Grafting
EuroSCORE: European System for Cardiac Operative Risk Evaluation
INR: International Normalized Ratio
AMT: Aortic Mural Thrombus
PAMT: Primary Aortic Mural Thrombosis
TEE: Transesophageal Echocardiography
CAD: Coronary Artery Disease
ILT: Intraluminal Thrombus
