# Supplementary material for: Repeated acute coronary syndrome caused by a mind-bending mural thrombus in ascending aorta: a case report and review of the literature
Source: BMC Cardiovasc Disord. 2024 May 29;24:281. doi: 10.1186/s12872-024-03956-2 (PMC11134645; doi:10.1186/s12872-024-03956-2)
Supplement: Supplementary file 1 — Supplementary Material 1 [file 12872_2024_3956_MOESM1_ESM.pdf]

Ultrasound Report

View Official Account   Explore cloud imagery

Medical Record Number: 112421369   Examination Number：US856439

Name: SXX   Gender: male   Age: 58 years old   Instrument: M9

Out-patient number: 20230103007750   Department: Emergency Department

Examination Area： [heart](Bedside)

Description

Cardiac function Assessment: LVEF 27%;
Color Doppler Flow Imaging (valvular regurgitation Assessment): Aortic valve: Trivial; Mitral valve: Trivial; Tricuspid valve: Trivial.
Left atrial inner diameter: 38mm
The myocardial thickness is within normal limits. There is diffuse hypokinesia of the left ventricular myocardium at rest. No significant effusion or separation is observed in the pericardial cavity.

Ultrasound Diagnosis

Decreased left ventricular systolic function with an ejection fraction of 27%, manifesting as diffuse hypokinesia of the left ventricular myocardium. Enlargement of the left atrium is also observed. Trivial regurgitation is present at the mitral , tricuspid, and aortic valves.

Date of Examination: 03-01-2023 23:53:23   Physician's Signature: 王悦
