# Supplementary material for: Repeated acute coronary syndrome caused by a mind-bending mural thrombus in ascending aorta: a case report and review of the literature
Source: BMC Cardiovasc Disord. 2024 May 29;24:281. doi: 10.1186/s12872-024-03956-2 (PMC11134645; doi:10.1186/s12872-024-03956-2)
Supplement: Supplementary file 2 — Supplementary Material 2 [file 12872_2024_3956_MOESM2_ESM.pdf]

绍兴市人民医院
Shaoxing People's Hospital
浙江大学绍兴医院
绍兴文理学院附属第一医院

超声检查报告单

关注公众号　　浏览云影像

病案号： 112421369　　　　检查号：US856439

姓名： 沈志云　　性别： 男　　年龄： 58岁　　仪器： M9

门诊号： 20230103007750　　科室： 急诊科　　病床：

检查项目：(特殊超声)心脏

超声描述(单位:mm)

床旁：

心功能估测：LVEF　27%
CDFI：瓣膜反流程度
主动脉瓣：少量
二尖瓣：　少量
三尖瓣：　少量
左房内径38mm

心肌厚度正常，静息状态下左室心肌活动弥漫性减弱。
心包腔未见明显分离。

超声印象

左室收缩功能减低（EF　27%），左室心肌活动弥漫性减弱；
左房饱满；
二尖瓣、三尖瓣、主动脉瓣反流（轻度）。

审核时间：2023-01-03 23:53:23　　记录员： 王悦　　审核医生：王悦

温馨提示： 请注意核对个人信息，此报告仅供临床参考，不作证明之用！
