# Supplementary figures and images for: Repeated acute coronary syndrome caused by a mind-bending mural thrombus in ascending aorta: a case report and review of the literature
Source: BMC Cardiovasc Disord. 2024 May 29;24:281. doi: 10.1186/s12872-024-03956-2 (PMC11134645; doi:10.1186/s12872-024-03956-2)

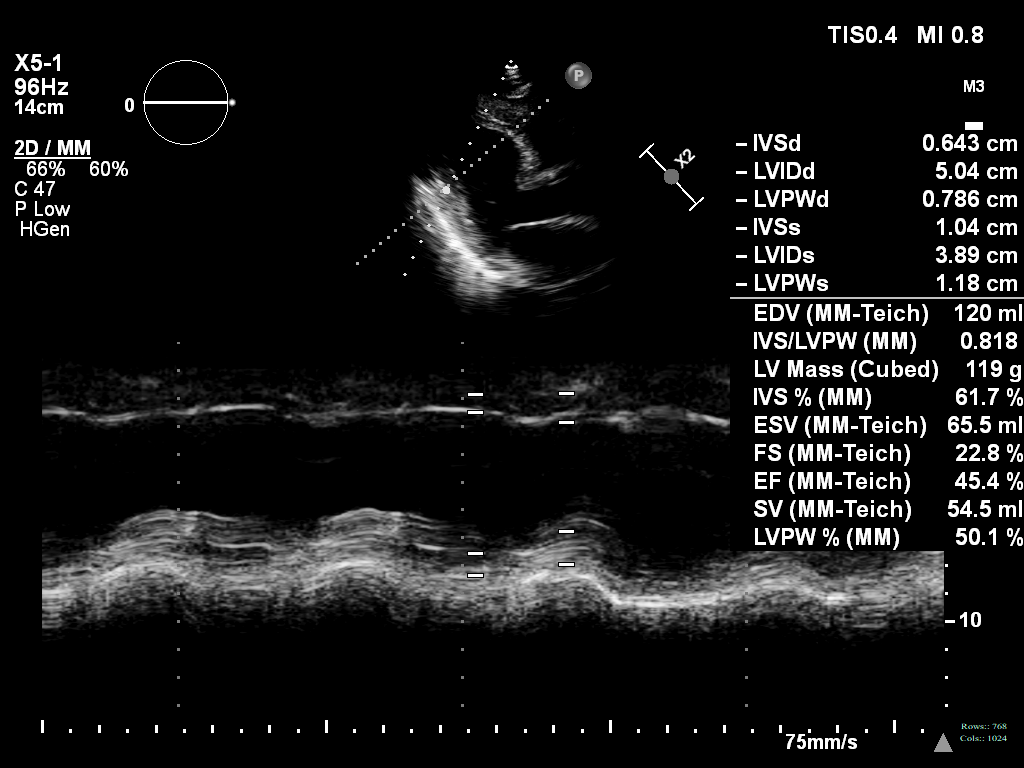

Supplement: Supplementary file 3 — Supplementary Material 3 [file 12872_2024_3956_MOESM3_ESM.tif]

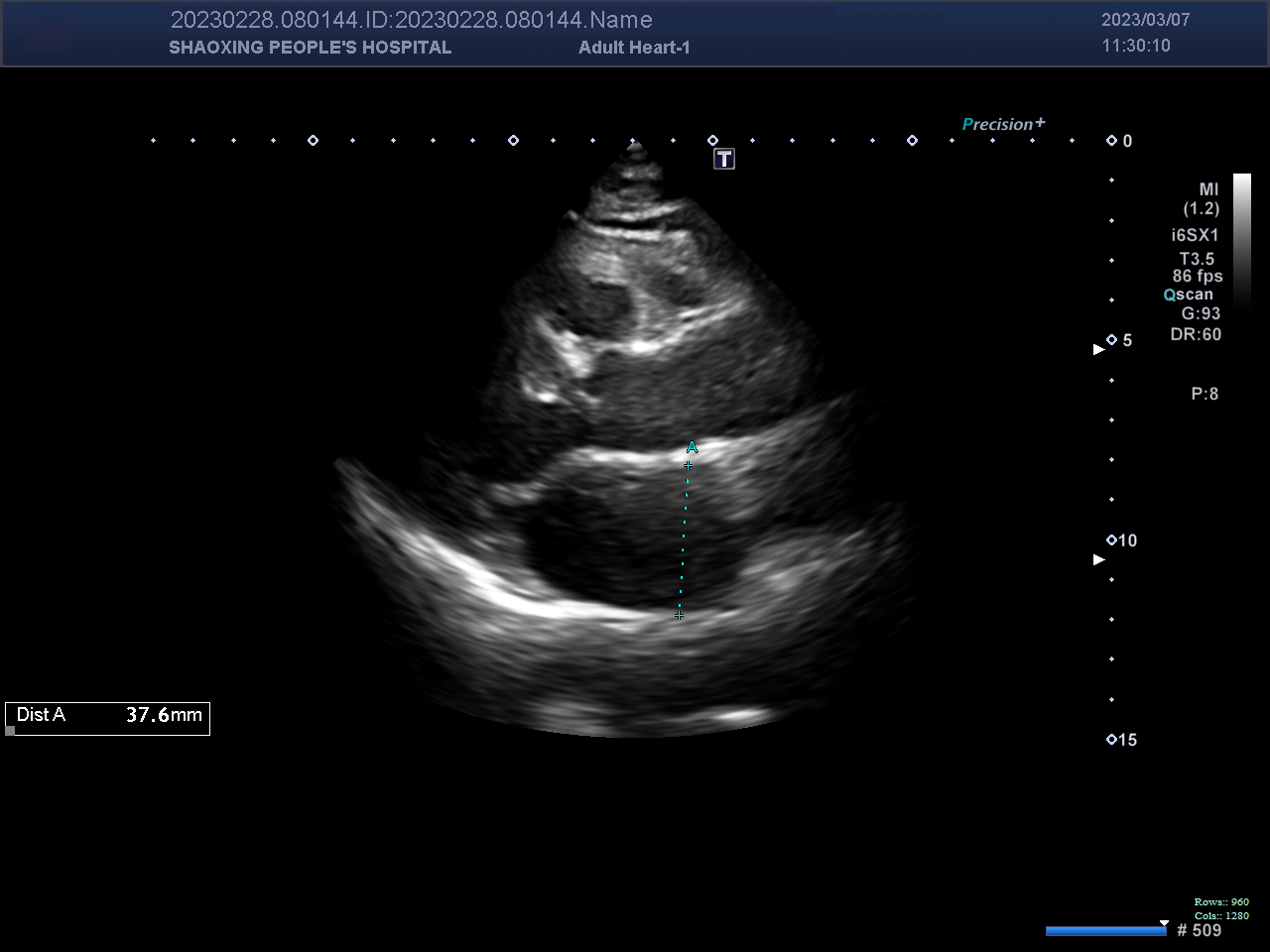

Supplement: Supplementary file 4 — Supplementary Material 4 [file 12872_2024_3956_MOESM4_ESM.tif]

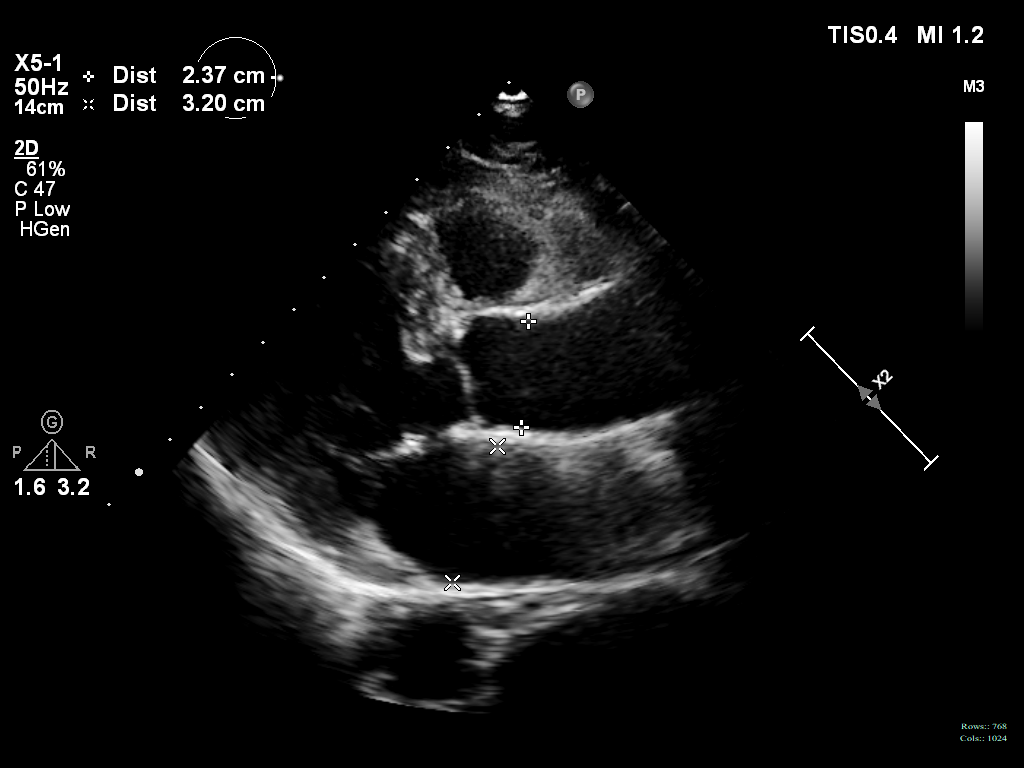

Supplement: Supplementary file 5 — Supplementary Material 5 [file 12872_2024_3956_MOESM5_ESM.tif]

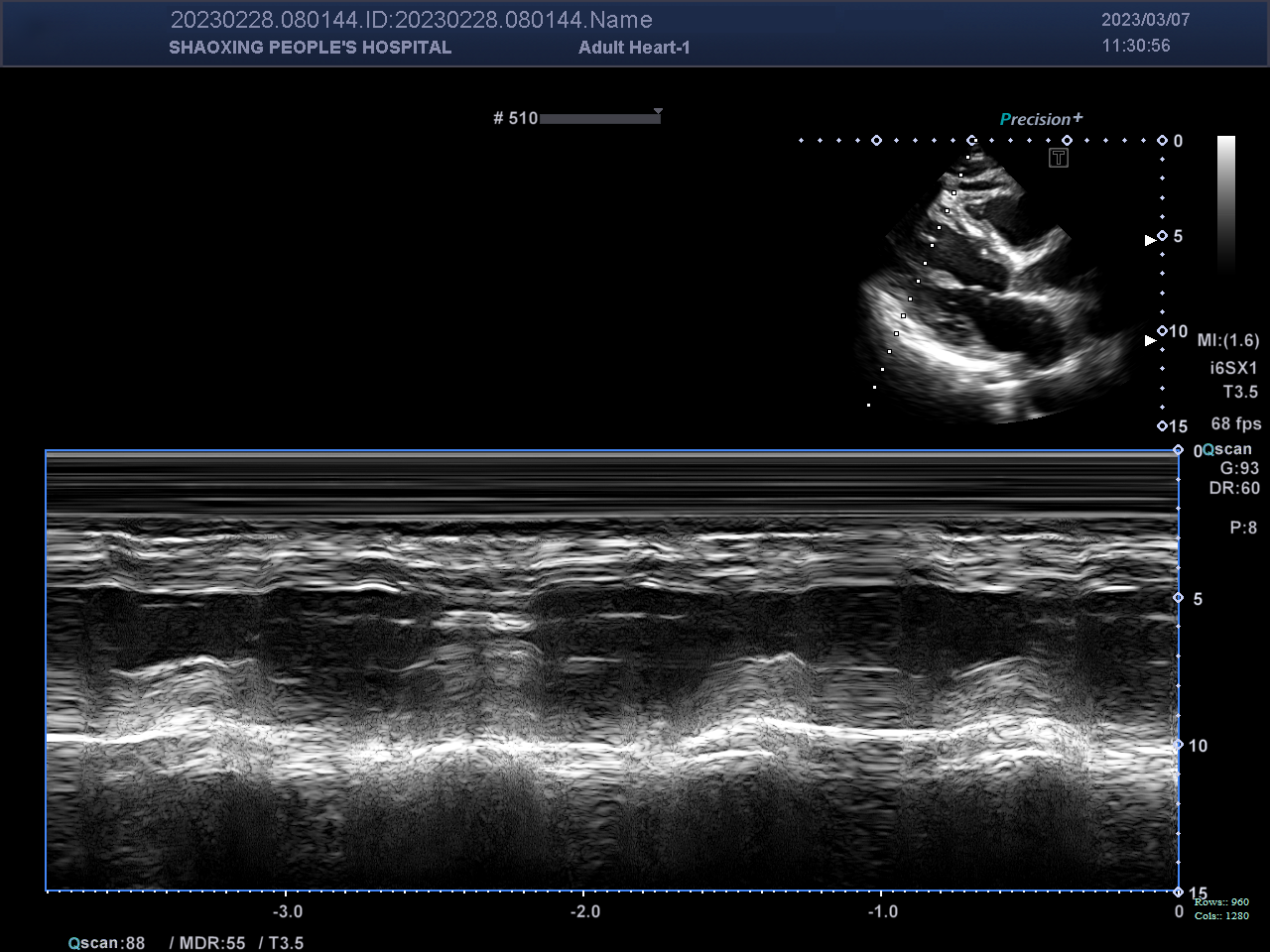

Supplement: Supplementary file 6 — Supplementary Material 6 [file 12872_2024_3956_MOESM6_ESM.tif]
